# Uncertain fate of pelagic calcifying protists: a cellular perspective on a changing ocean

**DOI:** 10.1093/ismejo/wraf007

**Published:** 2025-08-21

**Authors:** Adva Shemi, Assaf Gal, Assaf Vardi

**Affiliations:** Department of Plant and Environmental Sciences, Weizmann Institute of Science, Herzl St 234 7610001, Rehovot, Israel; Department of Science Teaching, Weizmann Institute of Science, Herzl St 234 7610001, Rehovot, Israel; Department of Plant and Environmental Sciences, Weizmann Institute of Science, Herzl St 234 7610001, Rehovot, Israel; Department of Plant and Environmental Sciences, Weizmann Institute of Science, Herzl St 234 7610001, Rehovot, Israel

**Keywords:** Coccolithophores, carbon cycle, ocean acidification, global warming, cellular calcification, algal blooms, Coccolith, Foraminifera, climate change, atlantification, calcite, carbon export, Gephyrocapsa huxleyi

## Abstract

Pelagic calcifying protists such as coccolithophores and foraminifera represent an important microbial component of the marine carbon cycle. Although their calcitic shells are preserved in oceanic sediments over millennia, their resilience in the future decades is uncertain. We review current literature describing the response of calcifying protists to ocean acidification and temperature warming. We examine these key ecological and biogeochemical processes through the cellular perspective, exploring the physiological, metabolic, and molecular responses of calcifying protists. Ocean acidification is a chemical process that takes place in the seawater outside the cell, whereas protists calcify inside a modified cellular microenvironment. The function of these calcification compartments depends on cellular response to ocean acidification, such as maintaining pH homeostasis. The response of calcifying protists to ocean acidification and temperature warming is species-specific, with no unifying trends but rather a range of sensitivity levels. Coccolithophores and foraminifera display physiological sensitivity that may hamper their ecological success in comparison to noncalcifying species. Yet, certain species may be more adaptable, especially when comparing to highly vulnerable calcifying molluscs as pteropods. As the molecular machinery mediating cellular calcification is not fully resolved, as well as the functional role of the calcitic shell, our ability to predict the fate of calcifying microorganisms in a warmer, more acidic ocean is limited. We propose the urgent need to expand the study of these model systems by advancing cell biology approaches and better understand the impact of climate change on microbial food webs in the ocean.

## Pelagic calcification in a changing ocean

The ocean is a sink for atmospheric carbon dioxide (CO_2_), which is being emitted in an unprecedented rate since the industrial revolution [1]. About 30% of the atmospheric CO_2_ is being absorbed by the seawater, leading to a significant increase in dissolved CO₂, which reacts with water to form carbonic acid (H_2_CO_3_), that dissociates into hydrogen (H^+^) and bicarbonate ions (HCO_3_^−^) [2]. This lowers the carbonate (CO₃^2−^) ion concentration [3] and the surface seawater pH, a process known as “ocean acidification” (OA) [1, 4]. The current atmospheric seawater CO_2_ concentration is ~425 parts per million (ppm) [5], with surface ocean pH of ∼8.1 [6]. By 2100, seawater CO_2_ is projected to rise to ~700–1000 ppm, which will drop ocean pH by ~0.1–0.4 units—more than 30% increase in acidity [6]. Furthermore, greenhouse gas emissions warm the atmosphere and are predicted to increase sea surface temperature (SST) by a global average of 2.6°C–4.8°C, with more frequent heat waves [6, 7]. Temperature warming alters water density, circulation, and stratification [8], which reduces nutrient availability [9]. Heat modulates seawater salinity, due to evaporation and sea ice melting [10]. These conditions remodel community composition, physiology, interactions, and biogeography of marine biota, including the “unseen majority” of microbes at the base of marine food webs, which impact oceanic nutrient cycles [11, 12].

Calcifying protists are microorganisms which contribute to the marine “Biological carbon pump” (BCP). The BCP mediates the drawdown of ~11 Pg C y^−1^ from the surface to the depth, by either the “Organic carbon pump” (for particulate organic carbon, POC) or the “Carbonate counter pump” (for particulate inorganic carbon, PIC) [13–16]. Calcifying protists, namely coccolithophores and foraminifera, produce PIC in the form of calcium carbonate (calcification, Ca^2+^ + 2HCO_3_^−^ → CaCO_3_ + CO_2_ + H_2_O). Coccolithophores (Haptophyta) are dominant phytoplankton forming blooms in the North Atlantic (NA) and North Pacific (NP) Oceans and in the Patagonian Shelf region [17–19]. They contribute ≤ half of the vertical CaCO_3_ flux in open waters [13, 15, 20] via their exoskeleton discs coined “coccoliths” [15]. Coccolithophore blooms stretch over thousands of square kilometers and composed mainly of the species *Gephyrocapsa huxleyi* [19] (synonym *Emiliania huxleyi* [21]). Planktic foraminifera (Rhizaria) are widespread unicellular heterotrophs or mixotrophs harboring photosynthetic symbionts [22], abundant in open waters such as the NA Ocean, and form spring blooms in diverse locations such as the Red Sea [23] and NP Ocean [24]. Their shells contribute up to 40% of the CaCO_3_ export to the depth [16]. The remaining 10% of the downward CaCO_3_ flux is contributed by the shells of free-swimming molluscs called pteropods [13]. Together, calcifying protists and pteropods generate up to 1.6 Pg CaCO_3_ y^−1^ in the pelagic zone [25]. Once buried in oceanic sediments, their shells are preserved for millions of years, allowing to deduce long-term trends of abundance and calcification, as well as fluctuations in environmental parameters that are being recorded in their shells [26, 27].

**Figure 1:**
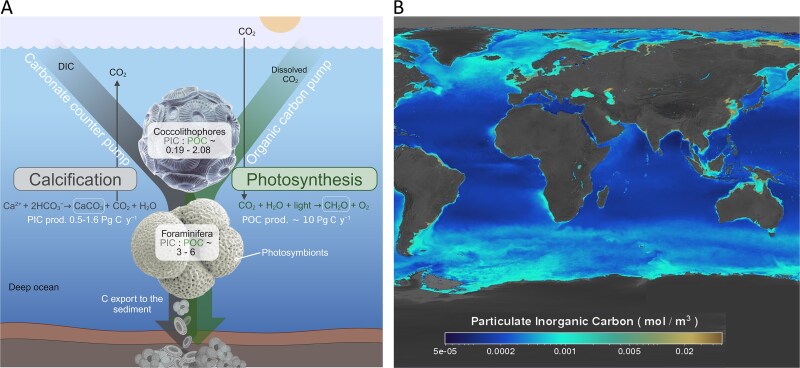
Calcifying protists and marine carbon sequestration. (A) The PIC:POC ratio links cellular carbon with marine carbon sequestration pathways. Coccolithophores and mixotrophic foraminifera fix CO_2_ via photosynthesis to produce particulate organic carbon (POC), which eventually sinks via the “Organic carbon pump.” In parallel, cells utilize dissolved inorganic carbon (DIC) to produce particulate inorganic carbon (PIC) in the form of CaCO_3_, stored in coccoliths and shells, which are exported to the sediment via the “Carbonate counter pump”. (B) A satellite image showing average PIC concentration (2002–2014). The Great Calcite Belt (GCB) appears as a bright ring around Antarctica. Image by NASA’s Goddard Space Flight Center.

Many biogeochemical studies link calcifying protists physiology with anticipated ecological impact by calculating their cellular PIC:POC balance under natural [17, 28, 29] or laboratory conditions [28, 30]. In coccolithophores and mixotrophic foraminifera, this ratio, known as the “rain ratio” (a “rain” of biogenic particles from the surface), estimates the relative cellular investment in biomineralization versus photosynthesis, where PIC:POC < 1 indicates a tendency toward lower calcification relative to photosynthesis, and PIC:POC > 1 indicates the opposite [31]. Planktonic foraminifera and coccolithophores have typical rain ratios in the range of 3–6 and 0.11–2.08, respectively [31–33] (Fig. 1A). A higher PIC:POC due to enhanced calcification may expedite sinking, as PIC is more dense than POC and therefore sinks faster [13]. Furthermore, POC is more labile than PIC, thus lower PIC:POC due to enhanced level of POC may imply on accelerated decomposition of suspended particles and thus lower carbon export to the sediment [13]. A fundamental question is how climate change will modulate the rain ratio of calcifying protists, which will have major consequences for carbon export? [4, 34, 35]. The depletion in carbonate ions reduces the saturation state of CaCO_3_ minerals in seawater, Ω_c_ (Ω_c_ < 1, where Ω_c_ is defined as the product of [Ca^2+^] × [CO_2_^−3^]/K, and K is defined as the stoichiometric solubility product) [36]; the increase in hydrogen ions decreases the ambient pH and may negatively affect cellular pH homeostasis and calcification [35]; the accumulation of CO₂ impacts photosynthesis; and the increase in bicarbonate ions influences both photosynthesis and calcification [37]. In coccolithophores, elevated temperature or increased CO_2_ decreases the rain ratio by more than a half due to reduced coccolith production [29, 38, 39] (Tables S1 and S2 and references therein). Incubation experiments examining prolonged adaptation [40] and model projections [35, 36] under different CO_2_ levels simulating OA further support these findings, implying on possible wide ecological consequences on future carbon sequestration by coccolithophores [36]. However, the impact of climate change on the rain ratio is complex, because calcification releases CO₂ rather than sequesters it, which is counteractive to the BCP and thus partially offsets its effect [13]. This phenomenon is investigated in zones with intense pelagic calcification such as the Great Calcite Belt (GCB) in the Sothern Ocean (~40–60S) [17]. The GCB comprises largely of coccolithophores and is clearly observed from space as turquoise watercolor due to the light scattered by the coccoliths [17, 41, 42] (Fig. 1B). Austral summer coccolithophore blooms contribute most of the PIC export south of the polar front [43] and have a high PIC:POC ratio [17]. These blooms enhance carbon sinking trough the BCP but may also lead to increase in seawater *p*CO_2_ during calcification, which counterbalances its drawdown by organic production and the strength of the BCP [13, 17].

**Figure 2:**
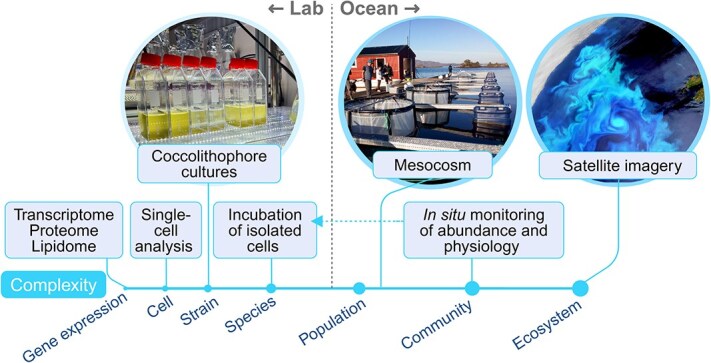
Approaches to investigating the response of calcifying protists to their environment. Experiments are conducted under increasing levels of ecological complexity, from the single cell to the ecosystem level. The satellite image shows a coccolithophore bloom with the characteristic milky color adapted from the European Space Agency (CC BY-SA 3.0 IGO).

The response of calcifying protists to ambient temperature or pH is studied at different levels of biological complexity, from the molecular basis to the ecosystem level (Fig. 2). Most of the knowledge gained is derived from short-term (days-weeks) experiments of isolated species and strains, which represent tractable, yet highly simplified experimental systems, and should be interpreted cautiously. Nevertheless, the expected changes in ocean chemistry are developed over time scales of years [4]. Hence, evolutionary experimental designs are useful to predict how contemporary protists will respond to future ocean conditions [40]. In addition, calcifying protists are monitored *in situ* and collected for morphological, metabolic, and genetic characterization [22]. Flow cytometry-based methods and single-cell technologies enable high-resolution analysis of environmental samples [44, 45]. The vast coccolithophore blooms are monitored by satellite imagery and floats equipped with bio-optical sensors which visualize PIC [17, 41, 46]. This ability to track a species (*G. huxleyi*, the most abundant coccolithophore [18]) by satellites and on the single cell level by flow cytometry is unique, allowing oceanographers to monitor traits over decades for the impact of climate change on *G. huxleyi* biogeography [19, 41], as well as to “hunt” blooms and study them in real time [47].

The current review employs a cellular perspective, exploring the physiological, metabolic, and molecular responses of calcifying pelagic protists to OA and ocean warming. First, we will zoom-in on the cellular mechanisms underpinning the rain ratio in calcifying protists in response to OA and temperature; then, we will zoom-out, discussing implications on long-term acclimation and food web interactions in marine ecosystems.

## Investigating the short-term cellular response of calcifying protists to temperature warming and OA

Temperature warming enhances metabolic rates, proliferation and photosynthesis in several coccolithophore species [33, 35, 48–50]. Most studies utilize *G. huxleyi* as a model, with available cultured ecotypes from diverse oceanic provinces [15, 30, 31] (Fig. S1). This species has a higher optimal growth temperature relative to other coccolithophores (~20°C, cell division of ~1 day^−1^) [50], suggesting a competitive advantage in response to marine heatwaves (Table S1 and references therein). A Red Sea *G. huxleyi* isolate exposed to heat treatment was more resilient and had faster adaptive growth response as compared to a co-occurring coccolithophore, *Gephyrocapsa ericsonii* [51]. At the molecular level, the proteomic response of *G. huxleyi* to heat stress includes reprogramming of photosynthesis and respiration pathways to optimize carbon usage [52]. Chloroplast-ribosomal proteins, photosynthesis, and chlorophyll synthesis proteins are downregulated, whereas heat shock proteins (such as Hsp90), mitochondrial, and TCA cycle components are strongly induced. The latter is probably promoted by an increase in acetyl-CoA production via pyruvate and acetoacetyl CoA [52]. The acceleration in metabolic rates and alteration in carbon metabolism are likely to impact cellular stoichiometry of carbon, nitrogen, and phosphorous [33] and enhance food web interactions and the efficiency of the BCP. Unlike coccolithophores, adaptation to warming is rarely studied in planktic foraminifera [53]. Temperature tolerance (measured as calcification rate) of *Amphistegina*, a benthic foraminifer from the Mediterranean Sea (SST 13–31°C) is higher than that of a sibling isolate from the Red Sea (SST 22–28°C) [54]. Similarly, in a thermally polluted coast in Israel, heat stress led to decreased shell volume of *Lachlanella* cells in comparison to an undisturbed control station [55]. Heat-tolerant species inhabiting this coast, such as *Pararotalia calcariformata*, can grow and calcify at 40°C [56] and display an altered chemical composition of their calcite shells [55]. These experiments highlight that heat resistance is species-specific, and that tolerant species may characterize key carbonate producers in future decades. This is crucial for Eastern Mediterranean ecosystem, where projected temperatures rise twice as fast than the global average [57]. The response of foraminifera can also be related to the impact of OA and temperature on their algal symbionts [58].

**Figure 3:**
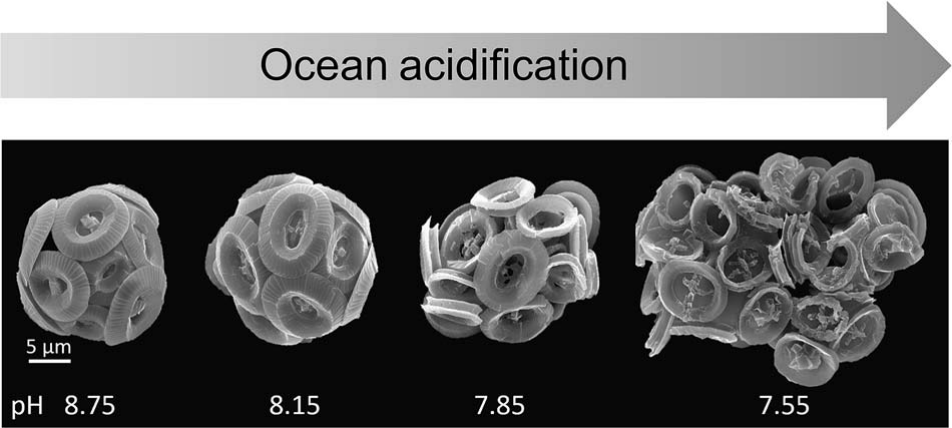
Coccolithophores’ response to acidified conditions in the lab. Representative cells from cultures incubated at different pH levels, with severe morphological defects following incubation in low pH. Modified from Kottmeier *et al*. [62], licensed under CC BY-NC-ND 4.0.

Growth, photosynthesis and calcification rates of *G. huxleyi* are fairly constant in response to mild OA conditions (~700 ppm CO_2_) over days-weeks (Table S2 and references therein). Some strains enhance photosynthesis, which may be attributed to an inefficient carbon concentration mechanism [2, 59], suggesting an advantage under elevated CO_2_ conditions. Higher levels of CO_2_ (950–1200 ppm, as expected by 2100 [60]) hinder proliferation and calcification in most strains (Table S2 and references therein). Sensitive strains lose their coccoliths [30], whereas more robust morphotypes continue to calcify [33, 61]. Nevertheless, a significant portion of the coccoliths observed is malformed [30, 61], also in heavily calcified species [62], displaying shortening of the shield elements [62], distorted symmetry and fragmentation [61] (Fig. 3). The coccolith hardness is attenuated, which may lead to impaired resistance against pathogens and grazers [63], as well as reduced cell ballast, which has consequences on carbon export to the ocean interior [13, 64].

The impact of OA on foraminifera is less studied than in coccolithophores. Foraminifera sequentially add calcareous chambers to the test covering the cell, and their growth is estimated by gouging shell size or quantifying the number of chambers precipitated per day (~0.1–1 day^−1^) [65, 66]. Planktonic foraminifera incubated in acid-treated seawater (pH = 7.8–7.5) have deficient growth, calcification, locomotion, and oxygen consumption, as compared with a control treatment (pH = 8.0–8.1) [34, 53, 65, 67]. The cells did not survive in pH 7.2. Among the species negatively affected by OA is *Neogloboquadrina pachyderma*, the main planktonic foraminifer in northern oceans [24, 53, 65, 68]. However, in a mesocosm study in the NA Ocean, CO_2_ enrichment did not have a clear impact on foraminiferal population [69]. Benthic foraminifera have suppressed growth and survival when incubated under OA conditions [66, 70] with reduced shell volume and weight, suggesting OA affects the quantity and quality of foraminiferal calcification [66, 70, 71].

## Elucidating the molecular machinery mediating cellular calcification and pH homeostasis in response to acidified conditions

The key to understanding the response of calcifying protists to OA is to reveal the subcellular architecture and molecular underpinnings of biomineralization. Coccolithophores are unique because they calcify intracellularly in a membrane organelle called the coccolith vesicle (CV), which delimits a very tight enclosure around the forming coccolith [72–74]. A genetic marker previously associated with calcification is GPA (glutamine, proline, alanine-rich protein), a protein which displays a Ca^2+^-binding activity, albeit its function remains unclear [75–77]. More recently, a suite of potential markers to assess calcification was suggested following comparative gene expression analyses of coccolithophore life stages (Table S3 and references therein). Most coccolithophores exhibit a haplo-diplontic life cycle, comprising diploid (2n) and haploid (1n) phases that can reproduce independently by mitosis. *G. huxleyi*’s diploid phase produces coccoliths, whereas the haploid phase is noncalcified [74, 78–80]. Specific proteins enriched in the diploid proteome control coccolith development in the CV, regulate the CV lumen pH (such as membranal V-type ATPase), and may mediate coccolith secretion (such as actin, myosin, and SNAP receptors -SNAREs) [76, 80, 81]. Carbonic anhydrases, which convert CO_2_ to HCO_3_^−^, are likely to be involved in calcification as well [77, 80]. Notably, adjusting cellular pH is critical in calcifying cells because CaCO_3_ formation from Ca^2+^ and HCO_3_^−^ results in the production of H^+^ that needs to be removed to prevent acidosis [79]. Ion exchangers in the plasma membrane play a fundamental role during calcification, including transmembrane HCO_3_^−^ and Ca^2+^ ion transporters and channels (i.e., Solute carrier 4 and cation/H^+^ exchanger (CAX) family, respectively) [75, 77, 80]. Additionally, H^+^/Na^+^ exchangers and a vacuolar-type ATPase also maintain pH homeostasis during coccolithogenesis.

**Figure 4:**
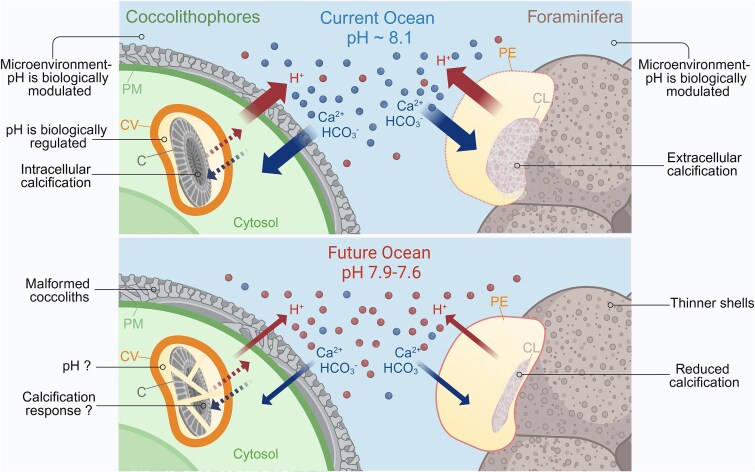
Ion exchange between the cell and the environment during pelagic calcification. Schematic representation of intracellular calcification in a coccolithophore, in comparison to extracellular calcification in a foraminifer. The coccolith (C) is formed in the coccolith vesicle (CV) in the cytosol, whereas in foraminifera, an external membrane cyst is formed at the site of the growing chamber. The calcification space is marked yellow. In both cases, active proton pumping (red arrows) and inward transport of carbonate ions (blue arrows) are needed to maintain cellular pH homeostasis. Dashed arrows represent ion fluxes maintained by putative channels in the CV. OA may interfere with the different ion fluxes, leading to malformed coccoliths and thinner shells. The effect of OA on the pH and calcification inside the CV is unknown, due to technical challenges to assess them *in vivo*. PM, plasma membrane; PE, protective envelope; CL, newly calcifying layer.

Previous molecular analyses under manipulated ambient pH, *p*CO₂ [37, 77, 82], Ca^2+^ [75, 83], or phosphate concentration [84, 85] (the latter was shown to induce calcification [84]), shed light on the molecular underpinnings cellular calcification. OA may disrupt the essential gradient needed for ion channels to operate, inhibiting proton pumping from the cell to the media (Fig. 4) [79].

Coccolithophores display different abilities to maintain pH homeostasis under OA conditions. In *G. huxleyi*, reduced calcification in response to OA was accompanied by induced expression of bicarbonate and ion transporters and channels [76, 86]. Cellular Ca^2+^ dynamics seem to be regulated by signal transduction pathways such as phosphatidylinositolphosphate kinases, sphingosine-1-kinases and associated downstream signaling kinases, i.e., calcineurin B-like proteins (CBL)-interacting protein kinases [86]. In *Coccolithus braarudii*, voltage-gated H^+^ channels in the plasma membrane regulate pH and allow calcification under normal conditions but have compromised activity in cells acclimated to low pH, leading to damaged coccoliths [62, 87] (Fig. 3). On the other hand, the coccolithophore *Ochrosphaera neapolitana* regulates pH homeostasis at the calcification site, unaffected by CO₂-induced changes in the ambient pH, by utilizing more dissolved CO_2_ rather than bicarbonate as inorganic carbon source [88]. This physiological adaptation allows *O. neapolitana* to calcify and photosynthesize under OA scenario (likewise, the PIC:POC ratio remains unaffected) [88]. Understanding the complex response of coccolithophores to OA is technically and conceptually challenging. In the CV, a nominal change by the import of two protons is equivalent to a pH reduction of 0.5, as the expected OA, yet there is no physiological meaning for the import of two protons into a vesicle. The implication is that instead of nominal concentrations, the process is regulated by ion fluxes in and out of the vesicle. It is likely that the balance between import and export proton fluxes is the actual chemical handle that affects the calcification process. In contrast to OA, warming homogeneously affects the cell and its environment, allowing for more direct correlation to the calcification process. Nevertheless, CaCO_3_ precipitation is more sensitive to pH changes than temperature; hence, most investigations are focusing on the effect of OA on the process.

Foraminifera calcify in an external privileged volume encased by a membrane (Fig. 4). Cellular pH homeostasis mechanisms are understudied in planktonic foraminifera, but in benthic species, a transient outward proton flux from the cell during chamber formation is maintained by V-type H^+^ ATPase [89]. Furthermore, high pH vesicles are transported to the calcification site [90], actively regulating pH in the cell microenvironment over a wide range of ambient CO_2_ levels.

## Examining long-term acclimation and microbial interactions of calcifying protists under climate change scenarios

Most studies investigating acclimation responses of calcifying protists include limited perturbation experiments using cultured isolates (Tables S1 and S2). However, the temporal and biological contexts of calcifying plankton in the changing ocean are significantly different. OA and climate change are processes that unfold over years. Moreover, calcifying protists are integral components of a complex microbial consortia, either as prey [91], predators [58], competitors for resources [92], and hosts for bacteria [93] and viruses [94, 95]. This disconnect creates a gap in our understanding of how calcifying protists will cope under future conditions. For instance, a mild increase in CO_2_ concentration did not change *G. huxleyi* cell growth in the lab (Table S2), but similar levels of CO_2_ inhibited coccolithophore bloom formation (with *G. huxleyi* as a dominant species) in several mesocosm studies [96–100].

### Long-term response of calcifying protists to changing pH and temperature conditions

In *G. huxleyi*, incubation for 700–2100 generations under OA conditions [40, 101], elevated temperature [102], or both [82, 103], revealed adaptable strains that maintain growth and may survive and acclimate to future ocean scenarios [40, 101, 104] (Tables S1 and S2 and references therein). Calcification response to prolonged OA or heat stress seems to be species-specific [40, 82, 101, 103]. Furthermore, populations grown under future ocean conditions for hundreds of generations exhibited a temperature optimum that is ~2.5°C higher than their ambient temperature, indicating adaptation or genotype selection [103]. Prolonged experiments are not available for foraminifera, which can be cultured only for short-term incubations.

Satellite imagery [42, 46], continuous plankton recording [105], sediment traps [22], and sediment cores [106] are oceanographic tools used to monitor calcifying protists’ abundance, diversity, dispersal and calcification trends [22]. Multidecadal increase in occurrence of coccolithophore populations was reported in the NA and NP Oceans, concomitantly with local rises in atmospheric CO_2_ and SST [105, 107, 108]. Moreover, “new” blooms were observed, for example, in the Black Sea during warm winters [46]. Albeit coccolithophores demonstrate high concentrations and a wider distribution in certain areas, low-latitude coccolithophores are predicted to be negatively impacted by climate change, with decreased growth rates, whereas other phytoplankton as cyanobacteria display improved growth rates [109]. Furthermore, bloom extent in the Bering Sea, in the NA Ocean south of Iceland, and in the Patagonian Shelf has diminished over the last decades [19], a decline associated with SST anomalies [19]. Models project a relative disadvantage of coccolithophore as compared to co-occurring phytoplankton taxa [92, 110, 111]. Together with their high inter- and intraspecific variability [51, 112, 113], these contrasting reports and predicts of coccolithophore success in future seas are inconclusive.

Planktonic foraminifera abundance is variable between seasons and years and is mainly controlled by temperature [23, 24], with some morphotypes associated with cold waters and others common in warm, tropic areas [114]. A recent global census of planktic foraminifera indicates their abundance and diversity decline, which is most pronounced in low- to mid-latitude regions [22]. Temperature and food availability, which is determined by local primary productivity, are driving this trend [24, 114, 115]. For example, in the subarctic Atlantic Ocean, surface current slowdown was hypothesized to reduce productivity and therefore planktonic foraminifera production [116]. Similarly, attenuated vertical mixing in the Mediterranean Sea inhibits primary production and thus foraminifera growth and diversity [22, 114].

**Figure 5:**
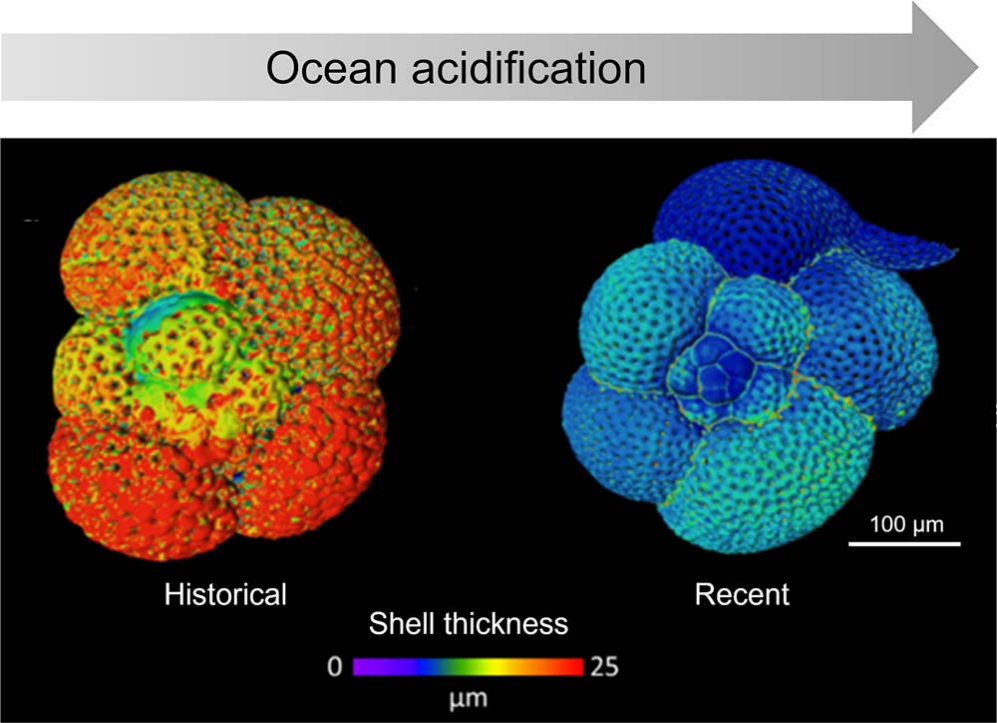
Ocean acidification may lead to thinner shells in modern foraminifera. Nanocomputed tomography reconstruction demonstrating shell thinning of wild foraminifera from recent and pre-industrial samples. Colors illustrate shell thickness, modified from Fox *et al*. [120].

Although abundances may show conflicting trends in different ocean basins [19, 107, 117], examination of calcareous shells or coccoliths reveals that calcifying protists are already been negatively affected by environmental changes. Comparative analyses from the Southern Ocean and Mediterranean Sea indicate that modern shells of coccolithophores [64, 106] and planktonic foraminifera [118, 119] are considerably lighter and smaller than those found in preindustrial (Holocene) sediments. Coccolithophore and foraminiferal shell weight loss was estimated as ~15–30% [64, 106] and ~10–40% [118, 119], respectively, depending on the species measured. The poor physiological state of modern planktonic foraminifera could also be deduced from comparative analysis of modern and historical (~1872) samples from the Pacific Ocean. Modern shells are up to 76% thinner than the historical samples, which theoretically could be explained by rising temperatures and acidification of modern oceans [120] (Fig. 5).

**Figure 6:**
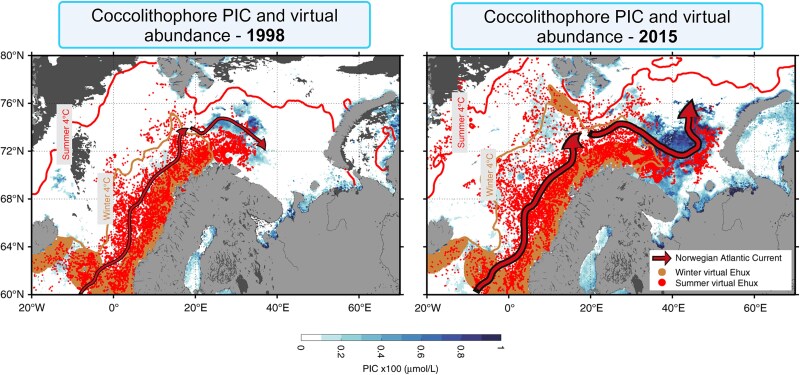
Poleward expansion of coccolithophores in the European Arctic corridor. Comparison between 1998 and 2015, where brown dots indicate inoculum of virtual particles in March, and red dots indicate their position in August. The particles drift with the current (arrows) as the ocean seasonally warms, as illustrated by the northward expansion of the 4°C isotherm. Remotely sensed PIC indicating coccolithophore biomass in summer (blue). The figure was modified from Oziel *et al*. [41].

Migration of calcifying plankton toward cooler habitats at higher latitudes is another long-term trend documented since the beginning of the century [22, 121]. Warming mediates the northward migration of coccolithophores [42, 122] and foraminifera [22], which were observed under-ice during the polar night [22, 123]. Advection is another mechanism facilitating migration of pelagic calcifiers. Recent attention was drawn to the “Atlantification” of the Arctic Ocean, caused by an increasing volume and velocity of a warm, salty current carrying North Atlantic coccolithophores as far as the northern Barents Sea (80–81N) (Fig. 6) [41, 124]. The high concentrations of Atlantic foraminifera and pteropods in the Arctic were also associated with this process [68, 125]. Another factor facilitating the migration of calcifying protists is human activity. The opening of the Suez Canal in 1869 allowed the invasion of Indo-Pacific benthic foraminifera into the Mediterranean Sea, a relocation exacerbated by climate change [126, 127]. Nevertheless, the current understanding of the adaptation of most pelagic calcifiers during the last decades suggests that lateral migration may not be a sufficient strategy in a changing ocean [22].

### Interactions of coccolithophores with pathogens and predators

Calcifying protists are integral in marine food webs as both prey and predators, yet we barely understand how OA or temperature may modulate their trophic interactions. Mesocosms, enclosures deployed in the environment where coccolithophores [29, 97] or foraminifera [69] commonly occur, allow to increase the abiotic and biotic complexity of the system and to gain ecologically relevant insights. As long as the function of coccoliths remains obscure, our ability to predict the consequences of OA on coccolithophores is limited [15]. If coccoliths serve as a “shield” against certain predators [63], their OA-mediated weakening might render coccolithophores to be more vulnerable [128]. In *G. huxleyi*, a pronounced mechanical damage to the coccolith following high CO_2_ treatment led to a 40% increase in copepod grazing rates [128]. Microzooplankton grazers (20–200 μm in size) are not deterred by the coccoliths [91] but may suffer from their inefficient digestibility, as their growth rate is compromised when consuming calcifying as compared to naked cells [129]. Therefore, OA could potentially lead to thinner coccoliths and easier uptake or ingestion of coccolithophores by different predators. OA and temperature warming may also alter trophic transfer efficiency, as they modify the cellular C:N ratio in a strain- and species-specific manner, making some cells less “nutritious” than others [49, 130, 131]. Temperature also modulates the saturation level of algal lipids that have both nutritional and signaling roles [132–134].

Climate forcing may impact coccolithophores resistance to pathogens. Blooms of coccolithophores are composed of *G. huxleyi* strains with various sensitivity to a lytic virus (*E. huxleyi* virus, EhV) [135], which is represented by a suite of virus genotypes with different degrees of virulence [136]. Host resistance can be acquired [135] by changes in ploidy [137] and lipid composition [138]. Lipids play essential roles during host–virus dynamics, as the virus is encapsulated with a membrane, synthesizes unique viral sphingolipids, and remodels the host lipid metabolism to fuel its replication cycle [132, 139, 140]. Considering viruses are fundamental mortality agents that terminate the bloom and expedite carbon export [141, 142], an important ecological question is how host–virus dynamics will be modulated in a warmer, more acidic ocean? In cultures, infection is inhibited in response to elevated temperature [95], which was linked to changes in lipid composition and membrane fluidity [139, 143]. Laboratory [144, 145] and field [98, 99] studies that examined the impact of OA on host–virus dynamics yielded conflicting results, with no clear trend of either the host or virus populations. Follow-up research is critically needed to better understand the effect of OA and temperature on coccolithophore–virus interactions. In recent years, pathogenic bacteria have emerged as a potentially important mortality agent during coccolithophore bloom termination [79, 146, 147]. Specifically, Roseobacters (α-Proteobacteria) can possess algicidal activity against *G. huxley*i [148, 149]. Elevated temperatures may induce the pathogenicity of *Ruegeria* toward *G. huxleyi* [93]. The ecological significance of this thermal effect on coccolithophore–bacteria interactions remains to be elucidated, as well as the completely unknown interactions of foraminifera with their potential pathogens.

### Photosymbiosis between foraminifera and dinoflagellates

The lifestyle of foraminifera comprises a spectrum of symbiotic states, ranging from strictly heterotrophic to various levels of photosymbiosis [71, 150]. Mixotrophic species are abundant and widespread, housing microalgae (such as dinoflagellates, diatoms, and others) which are transmitted during vegetative growth [58, 71, 151]. A single foraminifer can harbor 3000–20 000 photosynthetic cells, which together are able to photosynthesize and feed heterotrophically—an expanded metabolic capability which is advantageous in oligotrophic habitats. An example for the metabolic exchange between a foraminifer and it's dinoflagellate symbiont includes photosynthates translocated from the dinoflagellate to the host in the form of lipid droplets (soluble forms as amino acids are plausible as well) [58, 152]. Nitrogenous compounds are transferred from the digestive vacuoles of the host to the dinoflagellate, which displays a preference for ammonium over nitrate to fulfill its nitrogen requirements [153]. A planktonic foraminifer that inhabits oligotrophic tropical and subtropical seas loses its photosymbionts when incubated in acidified seawater [67]. However, the mechanism and ecological significance of this bleaching phenomenon and possible dysbiosis are understudied [67], as is the impact of climate change on foraminifera photosymbiosis in general.

Future work should inquire how OA and temperature modulate infection of calcifying cells by pathogens, their consumption by predators, as well as their mixotrophic lifestyle.

## Summary and discussion

Calcifying protists demonstrate a range of calcification and resilience states. Their biological response to OA and ocean warming is species-specific and complex, with limited physiological evidence available for planktonic foraminifera. Certain species or strains may acclimate to future ocean pH and temperature [65, 154], whereas others may perish or expand to new ecological niches where conditions are more suitable. These dynamics are expected to have implications for carbon export [13]. Nonetheless, the response calcifying protists in general to OA and temperature warming is more heterogeneous relative to pteropods, a dominant calcareous zooplankton in the Southern and Arctic Oceans [68, 155]. Pteropods form aragonite shells, a mineral polymorph of CaCO_3_ which is more soluble than calcite [13]. OA lowers aragonite saturation state more dramatically than it does for calcite, and therefore, pteropod are severely impacted by OA and warming, exhibiting reduced calcification [156, 157], shell dissolution [156, 158, 159], enhanced respiration and metabolism [160], and overall poor survival of adult [156, 161] and young life stages [162, 163].

### The current assessment of the impact of OA on cellular calcification is indirect

PIC weight and coccolith or shell morphology are routinely assessed to estimate calcification, but those parameters can only hint about the possible influence of OA on calcification. In addition to dissolution [66], OA interferes with the biogenesis of coccoliths and shells by disrupting proton gradients at the subcellular calcification site [79, 87]. This is challenging to monitor in coccolithophores, as the precipitation volume inside the CV is minute, and all chemical reactions happen in extreme confinement. It is difficult to directly link between ambient pH or cytosolic pH values to chemical perturbations that might affect the calcification process inside the CV.

### Calcifying protists may have divergent fates due to their different subcellular localization of calcification

The coccolithophore CV is more protected and isolated than the calcification sac of foraminifera, which is in contact with the environment, as exemplified by the uptake of dyes from seawater into the calcitic shell [164]. This fundamental difference may explain why certain coccolithophores are more resilient to environmental conditions [18, 165], whereas foraminifera are generally more sensitive [165]. Albeit acclimation has its cost in the form of reduced performance and calcite production, certain coccolithophores and foraminifera species may eventually adapt to increasing temperature and pH decline.

### Climate change is modulating the biogeography of coccolithophore and foraminifera on a global scale

In recent years, coccolithophore and foraminifera had expanded toward both poles [22, 121], yet it is unknown if they will sustain prolonged darkness or water freshening in high-latitude Oceans [10]. Because their migration is driven by the currents [41], it does not necessarily mean that coccolithophores will adapt to their new habitat at high latitudes (>80°) [124], with low solar radiation in late summer. Bloom dynamics may also be modulated by (new) interactions with the polar microbial community, such as microalgae and pathogens [94]. Succession of morphotypes may become common in regions highly impacted by climate change. Moreover, biomineralization is an energy-demanding cellular process, and the protective function of the coccoliths is not clear, suggesting tradeoffs between calcification and cellular stress response [63]. Phytoplankton communities could shift from coccolithophores to noncalcifying protists such as diatoms, dinoflagellates, and other picoeukaryotes [2, 92, 109].

## Future perspectives: advancing cell biology research in calcifying protists

### Developing direct markers to evaluate cellular calcification

Presently, the performance of calcifying protists is evaluated by measuring calcification endproducts, such as PIC weight, shell thickness, or coccolith morphology. Development of direct measurements of the subcellular biochemistry in the calcifying cell is mandatory in order to get mechanistic insights that will couple environmental changes to coccolith and shell formation. It is also critical to decipher the regulatory enzymes and functional macromolecules mediating cellular calcification [76]. Advancing our understanding of fundamental cellular processes will promote the development of sensitive tools to assess the impact of climate change on marine microbes.

### Investigating lipid remodeling in calcifying protists

In coccolithophores, the lipid production potential increases in response to OA and rising temperature, which may modulate their resistance to pathogens and benefit their consumers [49]. OA did not affect omega-3 polyunsaturated fatty acids, which are essential for heterotrophs’ diets. Yet species-specific modifications in phospholipids-derived fatty acids in response to OA were observed [130]. In response to elevated temperature, polar lipids such as glycosphingolipid are significantly induced [95]. As calcifying protists are food source for diverse consumers, it is important to understand how their lipidome will be modulated in the future. Furthermore, an important factor expected to be constrained by warming in planktonic cells is the degree of unsaturation (i.e., the number of double bonds) of fatty acids [133, 134]. In a cellular process called “homeoviscous adaptation”, an organism can alter the degree of lipid unsaturation to regulate the fluidity of the cell membrane in response to varying temperatures [133, 166]. Homeoviscous adaptation is understudied in calcifying protists and should be targeted for future biochemical studies, as it may have major implications on marine food webs and ultimately on fisheries [133].

### Entangling the complexity of natural coccolithophore and foraminifera populations by using high-resolution single-cell analyses and multiomics approach

High-resolution RNA sequencing, proteomics, and metabolomics are cutting-edge technologies that hold great promise for future research in the field. Single-cell analyses will provide a mechanistic understanding of the cellular response to external conditions and may reveal phenotypic heterogeneity [44], elucidating resilient subpopulations. Furthermore, rare species may play important ecological roles and should be utilized as models. For instance, *Coccolithus pelagicus* accounts for only 2% of the total coccolithophore community abundance in the Arctic Ocean but contributes more than half of the total regional calcite production (57%) [20], which is more than twice the contribution of the highly abundant *G. huxleyi* [20]. This suggests that rare species can be key players in the global carbon cycle under future climate change [20, 38].

### Connecting culture-based studies with field observations to provide a holistic view on calcifying microbes’ physiology and ecology

To understand the response of marine calcifiers to environmental change, laboratory and mesocosm experiments should be coupled with observational studies of biogeography. Future investigations must go beyond remote sensing and require isolations from the new bloom areas, such as the polar front [41]. Those isolates should be utilized for lab-based physiological and biochemical characterization, investigating their response to elevated CO_2_ and temperature, as well as subjecting them to diverse biotic interactions. Linking field and laboratory evidence will allow better understanding of mechanisms of acclimation and adaptation of calcifying protists in a changing ocean.

## Supplementary Material

supplementary_wraf007

## Data Availability

Data sharing is not applicable to this article as no datasets were generated or analyzed during the current study.
