# Supplementary material for: Uncertain fate of pelagic calcifying protists: a cellular perspective on a changing ocean
Source: ISME J. 2025 Aug 21;19(1):wraf007. doi: 10.1093/ismejo/wraf007 (PMC12573260; doi:10.1093/ismejo/wraf007)
Supplement: supplementary_wraf007 [file supplementary_wraf007.docx]

**
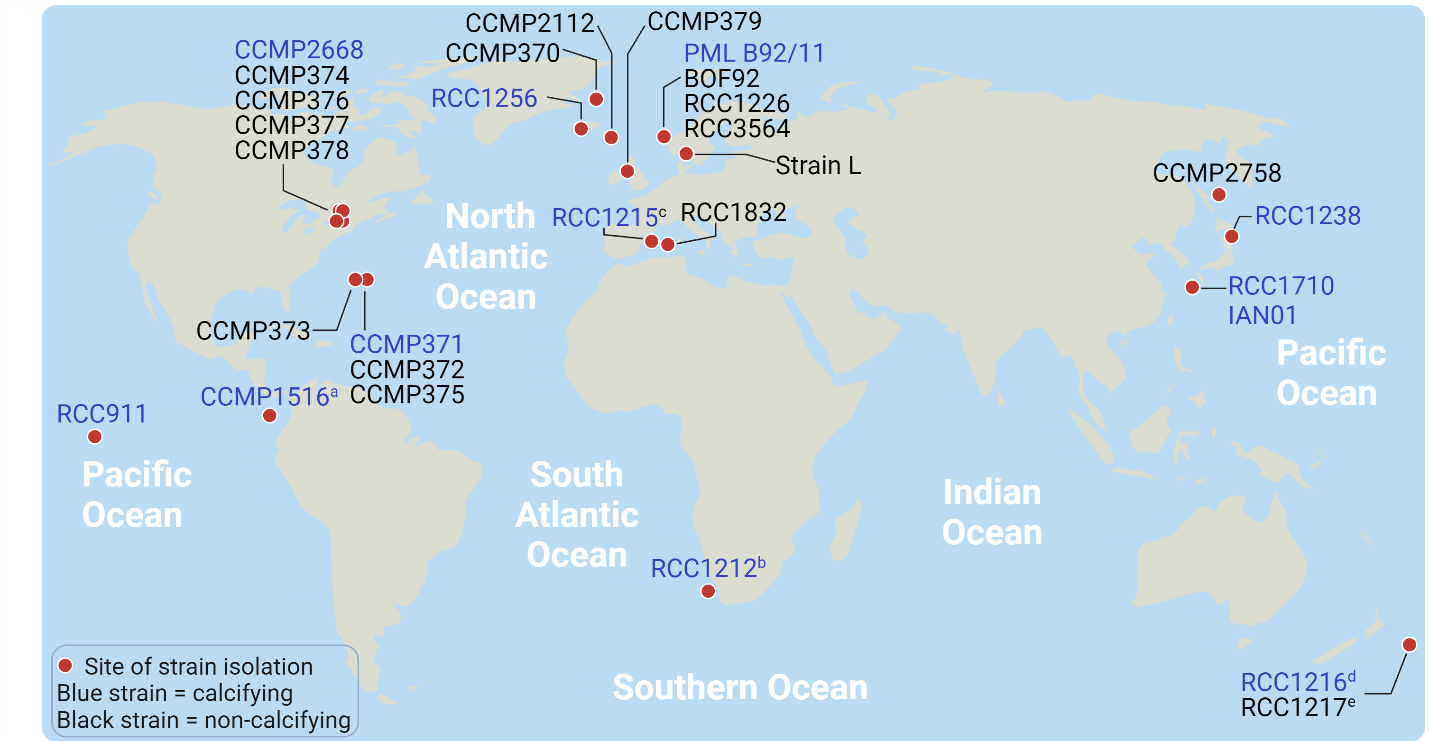
**

**Figure S1. The isolation sites of selected *G. huxleyi* cultures which are highly studied worldwide.** Studies focusing on the impact of OA and climate change on these strains are summarized in Tables S1 and S2. ^a^CCMP2090 is the axenic reisolate of this strain; ^b^synonym AC477; ^c^synonym TW1; ^d^synonym CCMP3266; ^e^synonym CCMP3268. Created with Biorender.com.

**Table S1. The physiological response of *G. huxleyi* strains to elevated temperature and combined temperature and CO_2_ stress under controlled laboratory conditions**. A summary of selected experiments testing the response of single strains to elevated temperature and elevated temperature+CO_2_ conditions for different durations (indicated as generations). Red arrow indicates an increase in the parameter tested, and blue arrow indicates a decrease. *Including photosynthetic efficiency measurements and POC production rates. PIC= particulate inorganic carbon; POC= particulate organic carbon; na= not assessed.


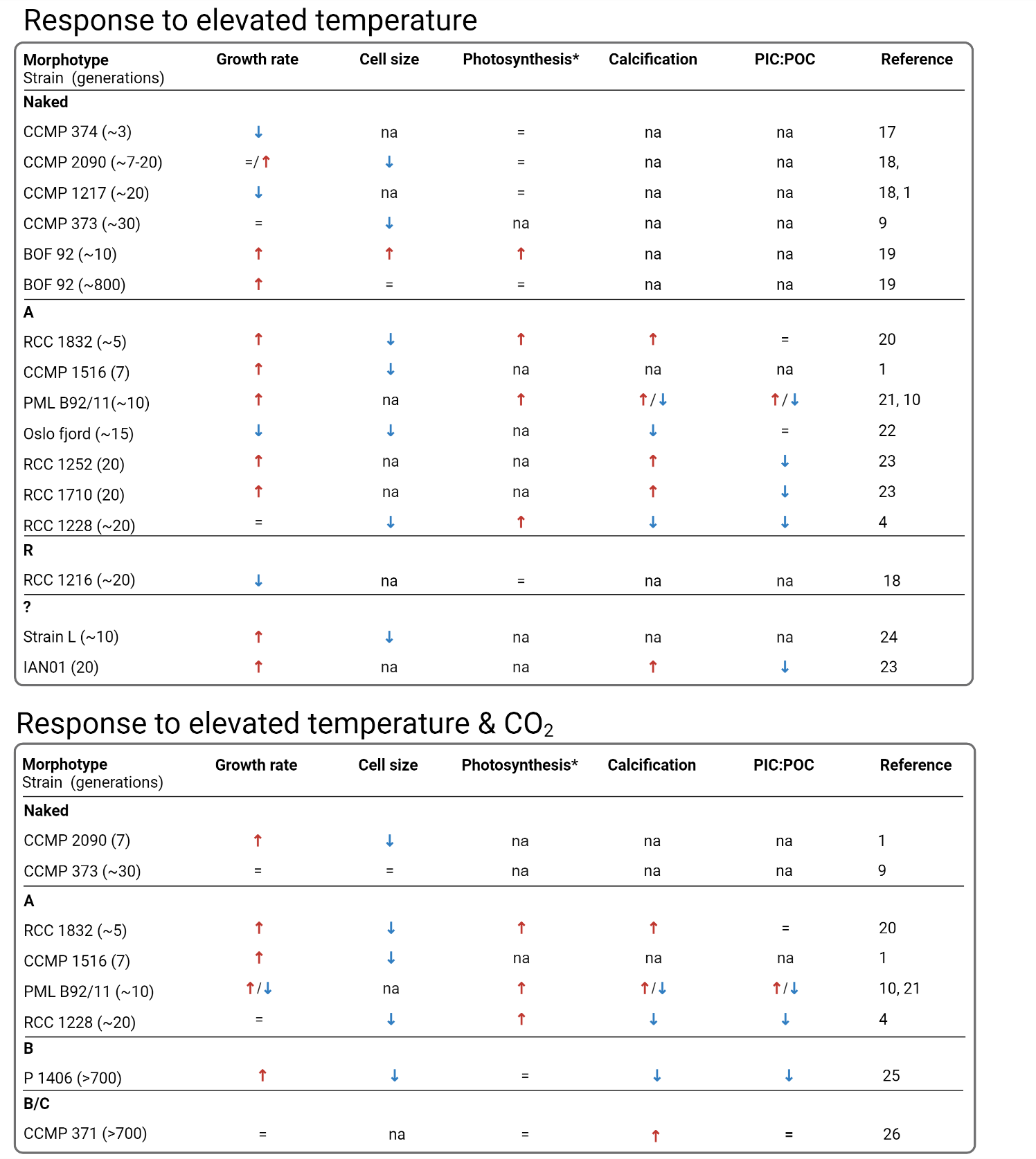


refernces [1-16]

**Table S2. The physiological response of *G. huxleyi* strains to elevated CO_2_ under controlled laboratory conditions**. A summary of selected experiments testing the response of a single strain to varying CO_2_ concentrations for different durations (indicated as generations). Red arrow indicates an increase in the parameter tested, and blue arrow indicates a decrease. *Including photosynthetic efficiency measurements and POC production rates. PIC= particulate inorganic carbon; POC= particulate organic carbon; na= not assessed. The “Bergen” isolates were isolated from independent mesocosms in Bergen.


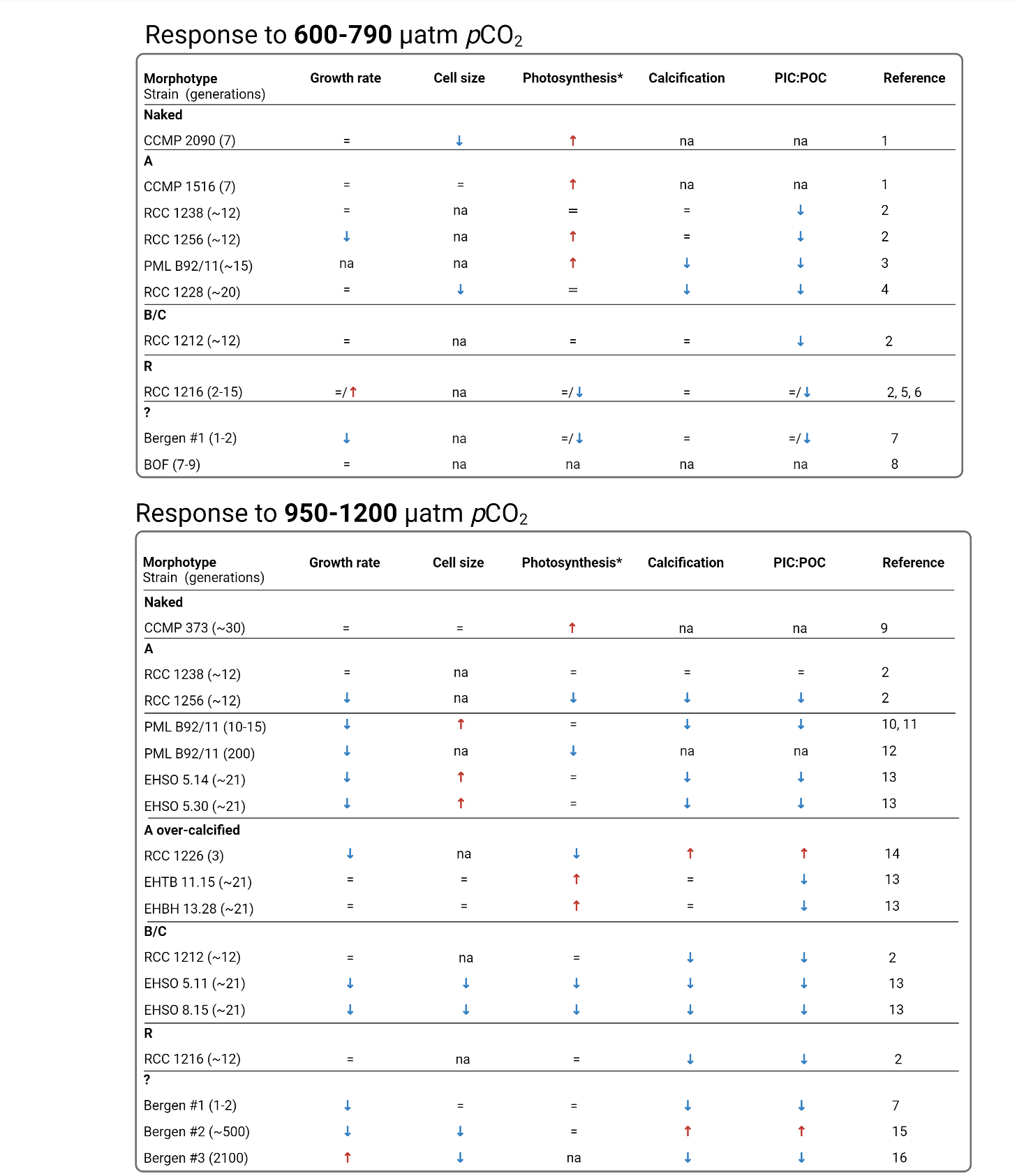


[17-26] [27-33]

**Table S3. Selected calcification-related proteins identified in *G. huxleyi.***

CV = coccolith vesicle.


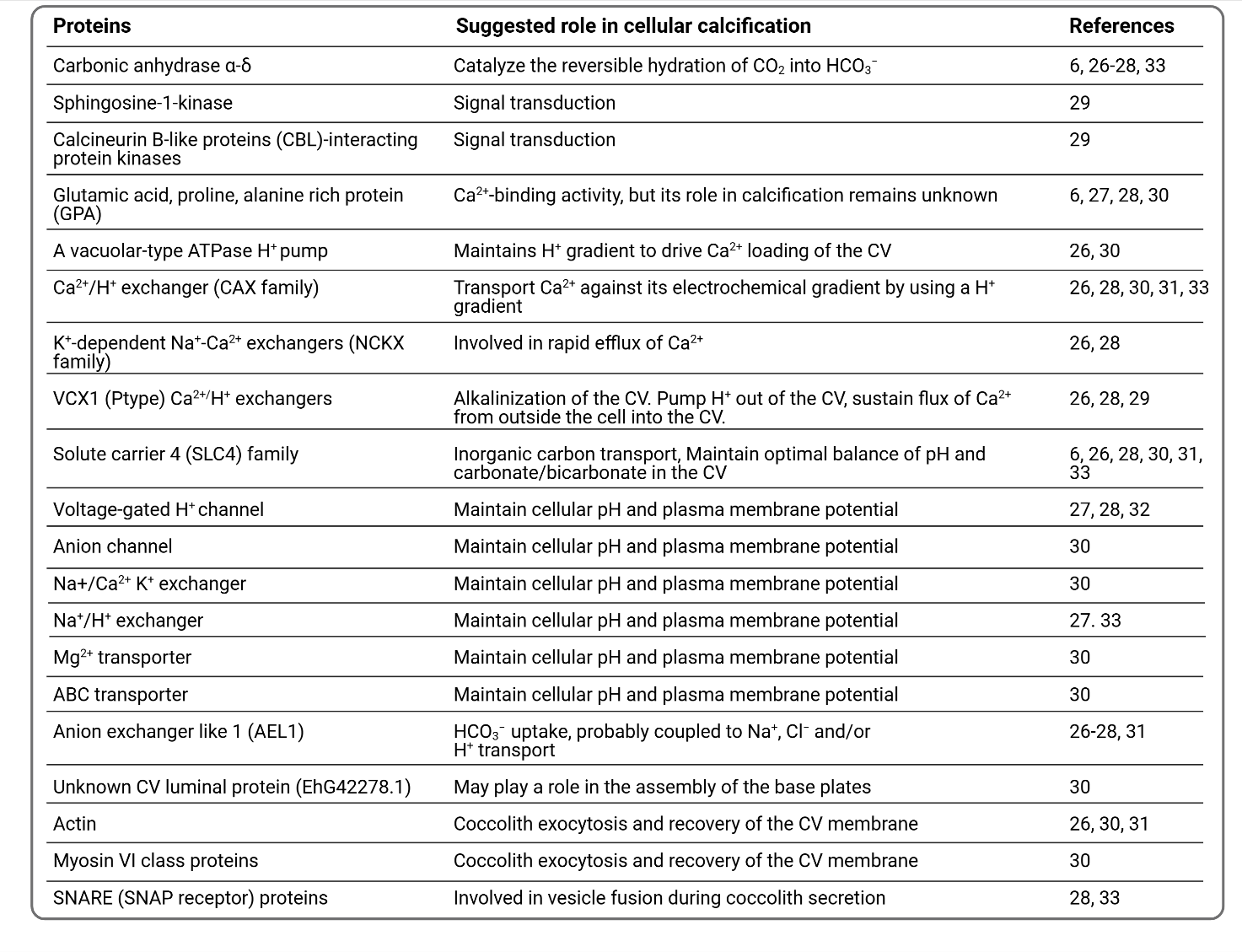


**Supplementary references**

1. Spielmeyer A, Pohnert G. Influence of temperature and elevated carbon dioxide on the production of dimethylsulfoniopropionate and glycine betaine by marine phytoplankton. *Mar Environ Res*. 2012;73:62-9. doi:10.1016/j.marenvres.2011.11.002

2. Langer G, Nehrke G, Probert I, Ly J, Ziveri P. Strain-specific responses of *Emiliania huxley*i to changing seawater carbonate chemistry. *Biogeosciences*. 2009;6(11):2637-2646. doi:10.5194/bg-6-2637-2009

3. Riebesell U, Zondervan I, Rost B, Tortell PD, Zeebe RE, Morel FMM. Reduced calcification of marine plankton in response to increased atmospheric CO_2_. *Nature*. 2000;407(6802):364-367. doi:10.1038/35030078

4. De Bodt C, Van Oostende N, Harlay J, Sabbe K, Chou L. Individual and interacting effects of *p*CO_2_ and temperature on *Emiliania huxleyi* calcification: study of the calcite production, the coccolith morphology and the coccosphere size. *Biogeosciences*. 2010;7(5):1401-1412. doi:10.5194/bg-7-1401-2010

5. Fiorini S, Gattuso J-P, van Rijswijk P, Middelburg J. Coccolithophores lipid and carbon isotope composition and their variability related to changes in seawater carbonate chemistry. *J Exp Mar Biol Ecol*. 2010;394(1):74-85. doi:https://doi.org/10.1016/j.jembe.2010.07.020

6. Richier S, Fiorini S, Kerros ME, von Dassow P, Gattuso JP. Response of the calcifying coccolithophore *Emiliania huxleyi* to low pH/high *p*CO_2_: from physiology to molecular level. *Mar Biol*. 2011;158(3):551-560. doi:10.1007/s00227-010-1580-8

7. Barcelos e Ramos J, Müller M, Riebesell U. Short-term response of the coccolithophore *Emiliania huxley*i to abrupt changes in seawater carbon dioxide concentrations. *Biogeosciences*. 2010;7doi:10.5194/bg-7-177-2010

8. Carreira C, Heldal M, Bratbak G. Effect of increased *p*CO_2_ on phytoplankton–virus interactions. *Biogeochemistry*. 2013;114(1):391-397. doi:10.1007/s10533-011-9692-x

9. Arnold HE, Kerrison P, Steinke M. Interacting effects of ocean acidification and warming on growth and DMS-production in the haptophyte coccolithophore *Emiliania huxleyi*. *Glob Change Biol*. 2013;19(4):1007-1016. doi:https://doi.org/10.1111/gcb.12105

10. Tong S, Hutchins DA, Gao K. Physiological and biochemical responses of *Emiliania huxleyi* to ocean acidification and warming are modulated by UV radiation. *Biogeosciences*. 2019;16(2):561-572. doi:10.5194/bg-16-561-2019

11. Yu J, Tian J-Y, Gao G, Xu R, Lai J-G, Yang G-P. Growth, DMS and DMSP production in *Emiliania huxleyi* under elevated CO_2_ and UV radiation. *Environ Pollut*. 2022;294:118643. doi:https://doi.org/10.1016/j.envpol.2021.118643

12. Xu H, Liu H, Chen F, Zhang X, Zhang Z, Ma J. et al. Ocean acidification affects physiology of coccolithophore *Emiliania huxleyi* and weakens its mechanical resistance to copepods. *Mar Environ Res*. 2023:106232. doi:https://doi.org/10.1016/j.marenvres.2023.106232

13. Müller M, Trull TW, Hallegraeff G. Differing responses of three Southern Ocean *Emiliania huxleyi* ecotypes to changing seawater carbonate chemistry. *Mar Ecol Prog Ser*. 2015;531doi:10.3354/meps11309

14. Vázquez V, León P, Gordillo FJL, Jiménez C, Concepción I, Mackenzie K. et al. High-CO_2_ levels rather than acidification restrict *Emiliania huxleyi* growth and performance. *Microb Ecol*. 2023;86(1):127-143. doi:10.1007/s00248-022-02035-3

15. Lohbeck KT, Riebesell U, Reusch TBH. Adaptive evolution of a key phytoplankton species to ocean acidification. *Nat Geosci*. 2012;5(5):346-351. doi:10.1038/ngeo1441

16. Schlüter L, Lohbeck KT, Gröger JP, Riebesell U, Reusch TBH. Long-term dynamics of adaptive evolution in a globally important phytoplankton species to ocean acidification. *Sci Adv*. 2016;2(7):e1501660. doi:10.1126/sciadv.1501660

17. Kendrick BJ, DiTullio GR, Cyronak TJ, Fulton JM, Van Mooy BAS, Bidle KD. Temperature-induced viral resistance in *Emiliania huxleyi* (Prymnesiophyceae). *PLOS One*. 2014;9(11):e112134. doi:10.1371/journal.pone.0112134

18. Mayers TJ, Bramucci AR, Yakimovich KM, Case RJ. A bacterial pathogen displaying temperature-enhanced virulence of the microalga *Emiliania huxleyi*. *Front Mar Sci*. 2016;7. doi:10.3389/fmicb.2016.00892

19. Zhou C, Zhang D, Yi X, Beardall J, Gao K. No adaptation to warming after selection for 800 generations in the coccolithophore *Emiliania huxleyi* BOF 92. *Front mar sci*. 2023;10doi:10.3389/fmars.2023.1211804

20. Johnson R, Langer G, Rossi S, Probert I, Mammone M, Ziveri P. Nutritional response of a coccolithophore to changing pH and temperature. *Limnol Oceanogr*. 2022;67(10):2309-2324. https://doi.org/10.1002/lno.12204

21. Sett S, Bach LT, Schulz KG, Koch-Klavsen S, Lebrato M, Riebesell U. Temperature modulates coccolithophorid sensitivity of growth, photosynthesis and calcification to increasing seawater *p*CO_2_. *PLOS One*. 2014;9(2):e88308. doi:10.1371/journal.pone.0088308

22. Gerecht AC, Šupraha L, Langer G, Henderiks J. Phosphorus limitation and heat stress decrease calcification in *Emiliania huxleyi*. *Biogeosciences*. 2018;15(3):833-845. doi:10.5194/bg-15-833-2018

23. Rosas-Navarro A, Langer G, Ziveri P. Temperature affects the morphology and calcification of *Emiliania huxleyi* strains. *Biogeosciences*. 2016;13(10):2913-2926. doi:10.5194/bg-13-2913-2016

24. van Rijssel M, Gieskes WWC. Temperature, light, and the dimethylsulfoniopropionate (DMSP) content of *Emiliania huxleyi* (Prymnesiophyceae). *J Sea Res*. 2002;48(1):17-27. doi:https://doi.org/10.1016/S1385-1101(02)00134-X

25. Armstrong E, Law CS. Resilience of *Emiliania huxleyi* to future changes in subantarctic waters. *PLOS One*. 2023;18(11):e0284415. doi:10.1371/journal.pone.0284415

26. Benner I, Diner RE, Lefebvre SC, Li D, Komada T, Carpenter EJ. et al. *Emiliania huxleyi* increases calcification but not expression of calcification-related genes in long-term exposure to elevated temperature and *p*CO_2_. *Philos Trans R Soc Lond B Biol Sci*. 2013;368(1627):20130049. doi:10.1098/rstb.2013.0049

27. Bach LT, Mackinder LCM, Schulz KG, Wheeler G,  Schroeder DC, Brownlee C. et al. Dissecting the impact of CO_2_ and pH on the mechanisms of photosynthesis and calcification in the coccolithophore *Emiliania huxleyi*. *New Phytol*. 2013;199(1):121-134. https://doi.org/10.1111/nph.12225

28. von Dassow P, Ogata H, Probert I, Wincker P, Da Silva C, Audic S. et al. Transcriptome analysis of functional differentiation between haploid and diploid cells of *Emiliania huxleyi,* a globally significant photosynthetic calcifying cell. *Genome Biol*. 2009;10(10):R114. doi:10.1186/gb-2009-10-10-r114

29. Rokitta SD, John U, Rost B. Ocean acidification affects redox-balance and ion-homeostasis in the life-cycle stages of *Emiliania huxleyi*. *PLOS One*. 2012;7(12):e52212. doi:10.1371/journal.pone.0052212

30. Skeffington A, Fischer A, Sviben S, Brzezinka M, Górka M, Bertinetti L. et al. A joint proteomic and genomic investigation provides insights into the mechanism of calcification in coccolithophores. *Nat Commun*. 2023;14(1):3749. doi:10.1038/s41467-023-39336-1

31. Mackinder L, Wheeler G, Schroeder D, von Dassow P, Riebesell U, Brownlee C. Expression of biomineralization-related ion transport genes in *Emiliania huxleyi*. *Environ Microbiol*. 2011;13(12):3250-3265. doi:https://doi.org/10.1111/j.1462-2920.2011.02561.x

32. Taylor AR, Chrachri A, Wheeler G, Goddard H, Brownlee C. A voltage-gated H^+^ channel underlying pH homeostasis in calcifying coccolithophores. *PLoS Biol*. 2011;9(6):e1001085. doi:10.1371/journal.pbio.1001085

33. Kao T-T, Lai M-W, Wang T-H, Yang C-L, Frada MJ, Ku C. Haplotype-aware multiomics unveils the regulatory basis of haplodiplontic life-cycle differentiation in a cosmopolitan marine alga. *bioRxiv*. 2024:2024.05.26.595999. doi:10.1101/2024.05.26.595999
